# Impacts of dual active-ingredient bed nets on the behavioural responses of pyrethroid resistant *Anopheles gambiae* determined by room-scale infrared video tracking

**DOI:** 10.1186/s12936-023-04548-9

**Published:** 2023-04-23

**Authors:** Katherine Gleave, Amy Guy, Frank Mechan, Mischa Emery, Annabel Murphy, Vitaly Voloshin, Catherine E. Towers, David Towers, Hilary Ranson, Geraldine M. Foster, Philip J. McCall

**Affiliations:** 1 Vector Biology Department, Liverpool School of Tropical Medicine, Liverpool, UK; 2 School of Engineering, University of Warwick, Coventry, UK

## Abstract

**Background:**

The success of insecticide treated bed nets (ITNs) for malaria vector control in Africa relies on the behaviour of various species of *Anopheles*. Previous research has described mosquito behavioural alterations resulting from widespread ITN coverage, which could result in a decrease in net efficacy. Here, behaviours were compared including timings of net contact, willingness to refeed and longevity post-exposure to two next-generation nets, PermaNet^®^ 3.0 (P3 net) and Interceptor^®^ G2 (IG2 net) in comparison with a standard pyrethroid-only net (Olyset Net™ (OL net)) and an untreated net.

**Methods:**

Susceptible and resistant *Anopheles gambiae* mosquitoes were exposed to the nets with a human volunteer host in a room-scale assay. Mosquito movements were tracked for 2 h using an infrared video system, collecting flight trajectory, spatial position and net contact data. Post-assay, mosquitoes were monitored for a range of sublethal insecticide effects.

**Results:**

Mosquito net contact was focused predominantly on the roof for all four bed nets. A steep decay in activity was observed for both susceptible strains when P3 net and OL net were present and with IG2 net for one of the two susceptible strains. Total mosquito activity was higher around untreated nets than ITNs. There was no difference in total activity, the number, or duration, of net contact, between any mosquito strain, with similar behaviours recorded in susceptible and resistant strains at all ITNs. OL net, P3 net and IG2 net all killed over 90% of susceptible mosquitoes 24 h after exposure, but this effect was not seen with resistant mosquitoes where mortality ranged from 16 to 72%. All treated nets reduced the willingness of resistant strains to re-feed when offered blood 1-h post-exposure, with a more pronounced effect seen with P3 net and OL net than IG2 net.

**Conclusion:**

These are the first results to provide an in-depth description of the behaviour of susceptible and resistant *Anopheles gambiae* strains around next-generation bed nets using a room-scale tracking system to capture multiple behaviours. These results indicate that there is no major difference in behavioural responses between mosquito strains of differing pyrethroid susceptibility when exposed to these new ITNs under the experimental conditions used.

**Supplementary Information:**

The online version contains supplementary material available at 10.1186/s12936-023-04548-9.

## Background

Resistance to insecticides has emerged in mosquitoes across the globe and threatens the future use of insecticides to control many vector-borne diseases. The most effective malaria control method in Africa, where the vast majority of malaria cases occur, is the widespread use of insecticide-treated bed nets (ITNs) [1] The first generation of ITNs use fast-acting pyrethroids, and pyrethroid resistance has spread rapidly through *Anopheles* populations in Africa [2-4] reducing ITN efficacy [5]. Several types of ‘next-generation ITNs’ are now available and used in many malaria-endemic countries; these all contain pyrethroids plus either an additional active ingredient (AI) with a different mode of action (MoA), or an insecticide synergist. Currently, the most widely used next-generation nets are pyrethroid-piperonyl butoxide nets (pyrethroid-PBO nets); PBO increases the potency of pyrethroids by blocking enzymes that break down these insecticides. In 2021, pyrethroid-PBO nets constituted 42.8% of the nets distributed in Africa with public funds [6]. Recent clinical trials of ITNs with two insecticides (Interceptor G2^®^, BASF, containing a pyrethroid plus the pyrrole insecticide chlorfenapyr) [7, 8] or containing pyrethroid plus pyriproxyfen (a chemical that sterilizes female adult mosquitoes) [9] have shown improved clinical outcomes (reduced malaria cases) over standard ITNs. However, improved epidemiological outcomes have only been demonstrated in a single setting with pyriproxyfen nets, showing no improved public health value over standard ITNs in the Tanzanian and Benin trials [7, 8]. Further evidence of their efficacy in different ecological and epidemiological environments is needed to support national ITN strategies.

The success of ITNs relies predominantly on aspects of the daily behaviour of the major malaria vectors in Africa, where *Anopheles* mosquitoes are largely anthropophagic, endophagic, endophilic and host-seek during the night when people are more likely to be underneath their bed nets [10, 11]. Multiple aspects of mosquito behaviour could change in response to widespread ITN use in ways that could decrease their efficacy [12, 13]. For example following a mass ITN distribution programme in Benin, one study suggested that *Anopheles funestus* have shown a shift in biting time, moving from a peak at 2am, to a peak in biting rate at 5am when people are likely to emerge from their protective ITNs [14]. Monitoring these population changes induced by the widespread deployment of ITNs, or any other vector control tool, is essential to explain and predict their epidemiological impact. Indeed, modelling studies have indicated that behavioural resistance and physiological resistance (caused, for example, by target site modifications or enhanced detoxification) could be equally detrimental to the efficacy of ITNs [12]. Therefore, surveillance of key vector behaviours should be an essential component of resistance management programmes.

In addition to population surveillance, critical insights into the behaviour of mosquitoes in response to ITNs can be gained by laboratory and semi-field studies that quantify important parameters. This includes net contact time and blood-feeding volumes and relates these to key endpoints such as longevity and reproductive outputs. Performing these tests on mosquito populations with different levels, and mechanisms of pyrethroid resistance may inform predictions on the efficacy of standard and next-generation ITNs in different environments. Standard WHO assays, designed to measure the performance of a single, fast-acting insecticide in ITNs (i.e., pyrethroids) are not suitable for measuring the impact of combining AIs with differing MoAs and endpoints. A series of benchtop and room-scale assays to record mosquito responses to a more diverse range of ITNs are currently in development and under evaluation.

The ‘baited box’ assay allows for close-range observation of mosquitoes attempting to take a blood meal through an ITN, with results from Hughes et al. [15], reporting that the accumulated duration of net contact by *Anopheles gambiae* was 50% lower on ITNs compared to untreated nets, with no difference in contact duration between susceptible and resistant mosquitoes [15]. Benchtop tests are undoubtedly informative, but the impacts of ITNs extend beyond the close range captured in these assays. Parker et al., [16, 17] used an infrared tracking system to characterize mosquito behaviour at mid-range, i.e., host-seeking events around an entire human-baited PermaNet^®^ 2.0 bed net (Vestergaard Sarl), from room entry to arrival at the ITN. The initial behaviour of insecticide-susceptible *An. gambiae* and wild *Anopheles arabiensis* did not differ between an untreated or pyrethroid ITN; mosquitoes continued to respond to the host without any evidence of repellency until they contacted the insecticide on the net surface. After this time, activity decayed rapidly, reaching zero after around 30 min, demonstrating the highly efficient rapid action of pyrethroid-treated ITNs. Here, this method is applied to studying the behaviour of insecticide-resistant mosquitoes to next-generation bed nets to gain initial insights into the utility of this method in comparing responses between mosquito populations and net types.

The present study compared the mosquito responses of pyrethroid susceptible and resistant mosquitoes to two next-generation nets, PermaNet^®^ 3.0 (Vestergaard Sarl) and Interceptor^®^ G2 (BASF AGRO B.V Arnhem [NL] Freienbach Branch), to a standard pyrethroid only ITN (Olyset™ Net, Sumitomo Chemical Co., Ltd) and to an untreated net. This study also sought evidence for any alterations in previously defined mosquito behaviours during host-seeking at the net, such as overall contact time, which may be attributed to the new nets.

## Methods

Mosquitoes from two insecticide-susceptible (Kisumu and N’gousso) and two insecticide-resistant (VK7 and Banfora) *An. gambiae *sensu lato (*s.l.*) strains were maintained under standard insectary-controlled conditions (27 °C ± 2 °C, and 80% relative humidity (RH)) at the Liverpool School of Tropical Medicine (LSTM). The susceptible *An. gambiae *sensu stricto (*s.s.*) Kisumu colony originates from Kenya (Shute, 1956) and has been maintained in colony since 1975. *Anopheles coluzzii* N’gousso was colonized from Cameroon in 2006 [18]. *Anopheles coluzzii* VK7 and Banfora strains originated from Burkina Faso, and have been reared at LSTM since 2014 and 2015, respectively, and are highly resistant to pyrethroids with susceptibility only partially restored by PBO pre-exposure [19, 20]. The VK7 strain is fixed for the knockdown resistant (*kdr)* 995F allele in the voltage-gated sodium channel (*Vgsc*), whereas the Banfora strain has a more complex set of *Vgsc* mutants [21]. Both strains have elevated cytochrome P450 expression, but additional resistance mechanisms are present in the Banfora strain including an increased metabolic respiratory rate [21]. All mosquitoes were reared under an altered 12:12 light/dark cycle to allow for testing to be conducted during the ‘night’ phase of the circadian rhythm.

The ITNs used are shown in Table 1. Nets were obtained directly from the manufacturer, aired at room temperature for four weeks prior to testing and then adjusted in size to fit the custom-made bed net frame, to eliminate creases and folds, ensuring maximum visualization of mosquito activity. A single net was used for each treatment, each stored at 4 °C between testing replicates (to ensure minimal net changes between replicates) and acclimatized at 27 ± 2 °C and 70 ± 10% humidity for at least 1 h prior to testing.

**Table 1:** Insecticide treated nets used in room scale tracking assays

Net type	Specification	Manufacturer
Polyester control	Untreated	Bayer AG, Leverkusen, Germany
Olyset Net (OL net)	150 denier polyethylene net incorporated with permethrin at 800 mg/m^2^	Sumitomo Chemical Company, Tokyo, Japan
PermaNet 3.0 (P3 net)	Roof: 100 denier polyethylene net incorporated with deltamethrin at 120 mg/m^2^ and PBO at 750 mg/m^2^, Sides: 75 denier polyethylene net with deltamethrin at 84 mg/m^2^	Vestergaard Sarl, Switzerland
Interceptor G2 (IG2 net)	75 denier polyester net coated with alphacypermethrin at 100 mg/m^2^ and chlorfenapyr at 200 mg/m^2^	BASF AGRO B.V Arnhem (NL), Germany

All experiments required a human volunteer to act as bait under the net. Volunteers were asked to wear light clothing, not to wear any strong scented products and not to bathe for at least 4 h prior to testing. During the experiment, volunteers were asked to lie as motionless as possible, while still being comfortable. To control for any effect of body positioning, volunteer orientation was randomly assigned either with head or feet nearest to the mosquito release point.

A total of 25, three-to-five-day old un-fed female mosquitoes were used per test replicate, as per Parker et al*.* [16]. Mosquito access to 10% sugar solution was removed by 16:00 the day prior to testing and replaced with distilled water; this was removed 3 h prior to testing.

### Experimental set-up

All experiments were performed in the LSTM Accelerator building, using a custom built free-flight testing room (7 m × 4.8 m in area, 2.5 m high) which is climate controlled (27 ± 2 °C and 70% ± 10% RH), while recording is operated from an adjacent room. Assays were performed during the afternoon to coincide with the ‘night’ phase of the mosquito’s circadian rhythm when they would be host-seeking in the wild. Frames made of carbon rods with roofs tilted towards the recording equipment were constructed for each bed net type to allow accurate observations of mosquito activity (dimensions: front height 45 cm, rear height 75 cm, roof width 90 cm, roof length 180 cm).

Mosquitoes were placed into a holding cup 1 h prior to testing to acclimatize within the testing room. The cup was attached to a long cord allowing mosquitoes to be released remotely by the operator outside the tracking room. Fifteen minutes before the test began the volunteer entered the ITN; to start the test, the release cord was pulled. After 2-h recording, free flying and knocked down mosquitoes were collected using a HEPA filter mouth aspirator (John. W. Hock, USA) to avoid any insect damage and placed into a fresh collection cup. Mortality was recorded at 24 h after test completion, with all mosquitoes individually monitored for sub-lethal insecticide effects (see below).

ITN treatments were changed approximately every three weeks and the testing room decontaminated between each ITN type, using 5% Decon90 solution (Decon Laboratories Conway Street, UK), followed by two water washes and a final wash with 70% ethanol. World Health Organization (WHO) cone tests [22] using susceptible *An. gambiae* were performed on the walls 24 h later to ensure proper decontamination. No such tests resulted in > 10% mortality [23], therefore all cleaning procedures were considered to remove any insecticide residue from the room. All room scale recordings were completed between June 2019 and February 2020.

### Mosquito tracking

Mosquitoes were tracked using paired identical recording systems, positioned 1050 mm apart and consisting of the following: each recording system used one camera (12 MPixel Ximea CB120RG-CM with a 14 mm focal length lens), aligned with a single Fresnel lens (1400 × 1050 mm and 3 mm thick, 1.2 m focal length; NTKJ Co., Ltd, Japan) placed approximately 1210 mm away. Cameras recorded with an exposure time of 5 ms and − 3.5 dB gain with a lens aperture of F#8.0 [24]. As experiments were carried out in the dark, infrared light was provided using custom ring light sources constructed by colleagues at Warwick university (12 OSRAM™ SFH 4235 infrared LEDs with a peak wavelength of 850 nm) which illuminated the total recording volume of 2 × 2 × 1.4 m. To reflect light back towards the cameras a custom designed Retroreflective screen (2.4 × 2.1 m, material: 3 M™ Scotchlite™ High Gain Reflective Sheeting 7610) was placed 2 m from the Fresnel lenses, with the bed and ITN placed in between both. The reflected light is focused by the Fresnel lens and forms a telecentric lens pair with an imaging optic mounted on the camera which allows illumination and imaging to occur from one side of the experimental set up. More information on signal processing can be found in Voloshin et al. [24]. Recordings were captured for both cameras over the 2-h assay using StreamPix recording software (StreamPix V7, Norpix, Montreal, Canada) at 50 frames per second (fps) onto a Windows PC (Intel^®^ Xeon^®^ Silver 4114 CPU 2.20 GHz, 24 Gigabytes RAM, Windows 10 Pro; 12 configured into 2 RAID arrays of 24 Terabytes each, at 1 array per camera.

### Video analysis

All video analysis was carried out using bespoke software written in Matlab (Mathworks) developed by collaborators at Warwick University [25]. Video segmentation, then compression to.mp4 files was performed before all videos were manually reviewed and cleaned to remove false tracks and human movement using ‘Sequential File Processing’ software [26]. Data extracted includes trajectory duration, distance travelled the number, duration and location of contacts with the bed net, time to first contact and track velocity, all of which have been previously described by Parker et al*.* [16]. Additional track joining and the deletion of false tracks created by volunteer and camera noise was performed in ‘Post Processing’. Activity was categorized into behavioural modes (Table 2) using existing quantification algorithms [26] and recorded as occurring in one of ten non-overlapping regions of the bed net. Since many mosquitoes were released into the room in all tests, tracking individual mosquitoes was not possible, hence analysis was performed on flight tracks with each track from entry into and exit out of the field of view analysed separately. One flight track could consist of three different behavioural modes (visiting, bouncing and resting as they all involve net contact), upon which the time spent in each mode were recorded separately.

**Table 2:** Definition of mosquito behavioural modes (adapted from [17])

Behavioural mode	Definition
Swooping	Flight tracks without net contact
Visiting	Tracks where extended periods of flight were interspersed with infrequent contacts with the bed net. Contacts were characterized as sharp 80° turns or more in the trajectory, and when multiple contacts occurred with the net, the minimum interval between each contact was 0.4 s (i.e., an interval of at least 20-frames, at 50 frames per second)
Bouncing	Tracks where the mosquito made multiple contacts at intervals of less than 0.4 s with the bed net surface; including tracks with short flights between the contacts, or tracks maintaining contact with the bed net surface without being static. This includes ‘walking’ or ‘probing’ the net with gaps in movement lasting less than 0.75 s
Resting	Tracks where the mosquitoes were static for at least 0.75 s on the net surface, or where the velocity of mosquito movement was less than 1.33 mm/s. Mosquitoes were classified as dead and excluded by limiting resting periods to a maximum of 300 s. No dead mosquitoes were found on nets at the end of any test

### Life history traits

**Fig. 1:**
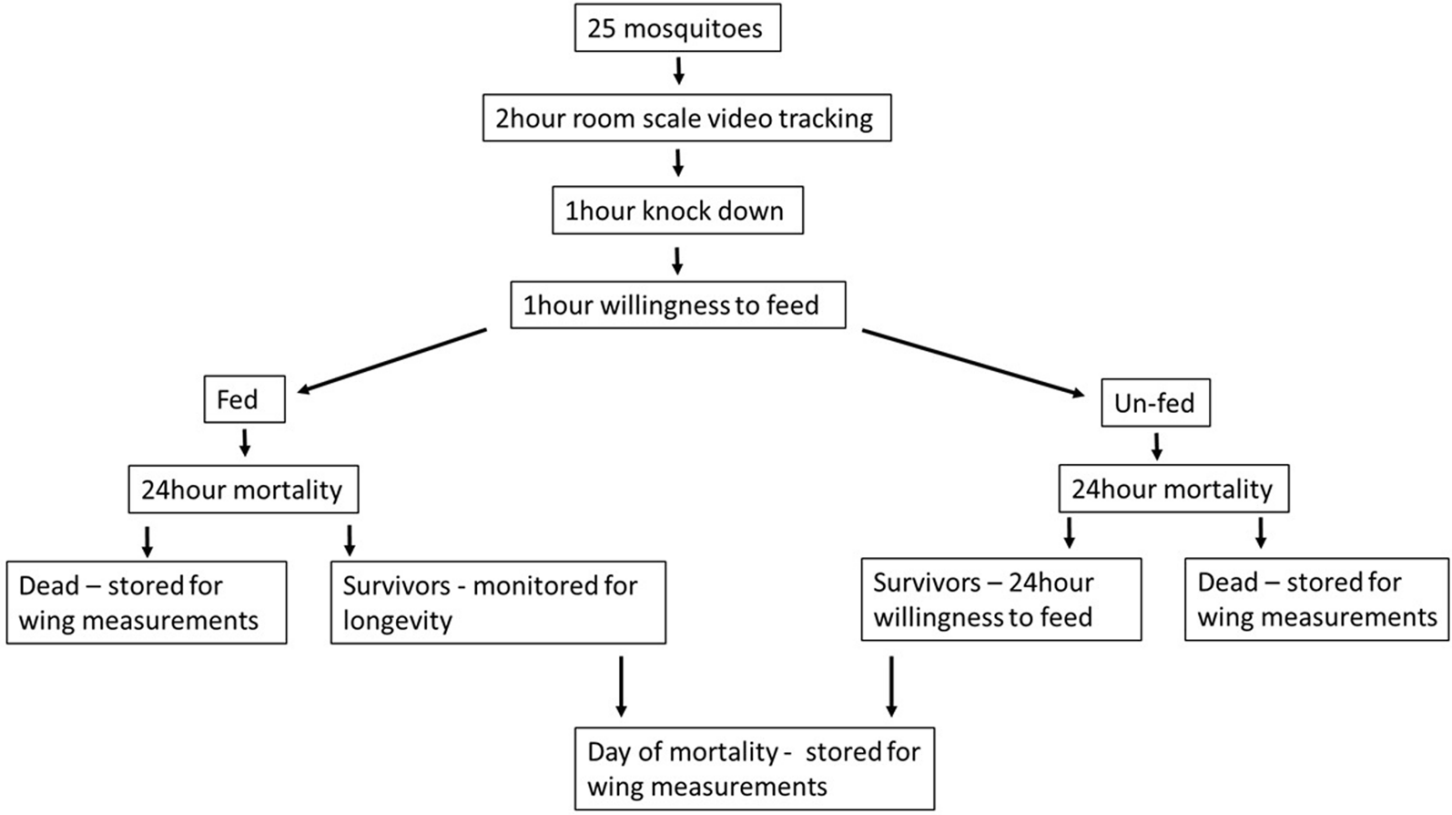
Measured sub-lethal pipeline outcomes per room scale video tracking assay

The methods monitoring life history traits have been previously described in Hughes et al. [28]. After each tracking assay, the following were measured for each mosquito: 24-h mortality, willingness to feed at 60 min, or 24 h (by exposure to the arm of a human volunteer), longevity and wing length (Fig. 1).

### Data analysis

In this pilot study, a target of six replicates was set for each mosquito strain/ITN combination, based on previous studies [17]. Volunteer effect was not investigated in this study.

### ITN bioefficacy and mosquito longevity

Bioefficacy of nets was assessed through measuring mosquito mortality post-exposure. Mosquitoes were transferred to individual falcon tubes, provided with a source of 10% sugar water and mortality measured daily until all mosquitoes had died.

### Quantifying mosquito activity and behaviour

Total activity for each strain (seconds of movement) and net treatment was calculated as the sum of all mosquito activity, regardless of behavioural mode and binned into 5-min intervals for analysis. Further analyses were performed using the total activity stratified into the four described behavioural modes (swooping, visiting, resting and bouncing).

### Defining and quantifying mosquito contact with the bed net interface

Total number of contacts with the net and total contact duration were calculated from the sum of all contacts obtained from visits, bounces or resting tracks. Total duration of contact in the first 10 min of the assay was calculated as a percentage of total contact duration for the entire replicate. As it was not possible to determine individual mosquito contact, the possible minimum and maximum values of net contact were calculated, as in Parker et al. [16]: for the maximum value, total contact duration was divided by the maximum number of mosquitoes seen simultaneously contacting the net in one frame of the recording (recording is performed at 50 frames per second); the minimum value assumed that all 25 mosquitoes released into the assay responded at the same time.

### Determination of contact location

The recording field of view was divided into 16 regions as previously described [26]. Ten of these regions were on the net surface; six on top of the bed net, two on the front of the net and one at either side.

### Speed around the bed nets

Flight speed was analysed using whole swooping tracks around the bed nets to investigate any changes in mosquito free-flying speed away from the bed net.

### Mosquito activity decay over the 2-h assay

Exponential decay modelling was considered for analysis of activity over time, as reported previously by Parker et al. [16] but many of the test replicates violated the equation constraints, so an alternative method was used whereby total activity in the first 5 min of recording was subtracted from total activity in the final 5 min of recording. A negative value indicated that activity decayed over time and a positive value represented an increase in activity between the two timepoints.

### Determining willingness to refeed and mosquito size

Wing length was used as an estimate for mosquito body size and to control for potential size differences between cohorts [28]. The right wing was removed, and an image taken using GXCAM ECLIPSE Wi-Fi camera attached to a GX Stereo microscope (GT Vision Ltd). The length of the wing was measured from the axial vein to the distal end of the R1 vein using GXCAM software (GXCAM Ver.6.7).

To assess effects of sub-lethal insecticide exposure, surviving mosquitoes were offered a blood meal (a human arm placed over a netted cup) at 1-h post-exposure and subsequent longevity monitored. Blood feeding inhibition was calculated by considering all mosquitoes in each replicate and assessing whether they were willing and able to take a blood meal or not.

### Statistical analysis

Statistical analysis was performed used Prism 6 (GraphPad) and R (Version, 1.1.463, R Core Team 2019). 24 h mortality was assessed using t-tests for the comparison of observed means, and mosquito longevity was analysed using Kaplan Meir Long-rank (Mantel-Cox) tests. Shapiro–Wilk tests were carried out on all activity data to check for normality. Total activity was analysed used Welch’s ANOVA as it was not assumed that all groups sampled were from populations with equal variance. Generalized linear models (GLMs) with normal probability distribution were used to analyse pairwise comparisons of mosquito strain and net type for: behavioural mode, contact number, contact duration, duration of contact in first 10 min, average contact duration, swooping speed, activity decay, willingness to refeed and wing length. Post-hoc analysis used the Tukey method of adjustment for comparing a family of four estimates. A binomial GLM was used to look for any interactions that might explain a relationship between net contact duration and mortality, however the model showed that there was no interaction between net type and contact duration or strain and contact duration. A GLM was performed to investigate the relationship between mosquito wing size and blood feeding success, considering interactions with mosquito strain and net type. For all statistical comparisons, the α threshold used was 0.05. Unless stated otherwise, 95% confidence intervals are reported.

### Ethical permission

With no infection risk and no exposure to untested chemicals, the procedures involved in generating these data results did not require clearance by LSTM Research Ethics Committee. Written consent was obtained from all volunteers.

## Results

A total of 1690 mosquitoes was tested across 73 assays, with 18 different volunteers being used as a human ‘bait’. The total number of replicates performed for each strain and treatment is shown in Table 3. It was not possible to reach the target replicate number of six for all strain and net treatment combinations because several video files were corrupted during a computer failure resulting in missing videos. Replacement replicates could not be done for PermaNet 3.0 and Interceptor G2 nets due to national COVID-19 restrictions and the loss of high level pyrethroid resistance in the LSTM Banfora colony.

**Table 3:** Total number of test repeats performed per ITN, per mosquito strain

Strain	Recording dates	Long-lasting insecticidal nets			
		UT net	OL net	P3 net	IG2 net
Kisumu	Jun 2019–Jan 2020	5	6	6	6
N’gousso	Jun 2019–Nov 2019	4	6	2	6
*VK7*	Jun 2019–Feb 2020	4	5	5	5
*Banfora*	Jul 2019–Dec 2020	4	6	3	0

Resistant mosquito strains are denoted in italics

*UT *untreated net, *OL* Olyset Net, *P3* PermaNet 3.0, *IG2* Interceptor G2

### Mosquito survival

#### Bioefficacy

**Fig. 2:**
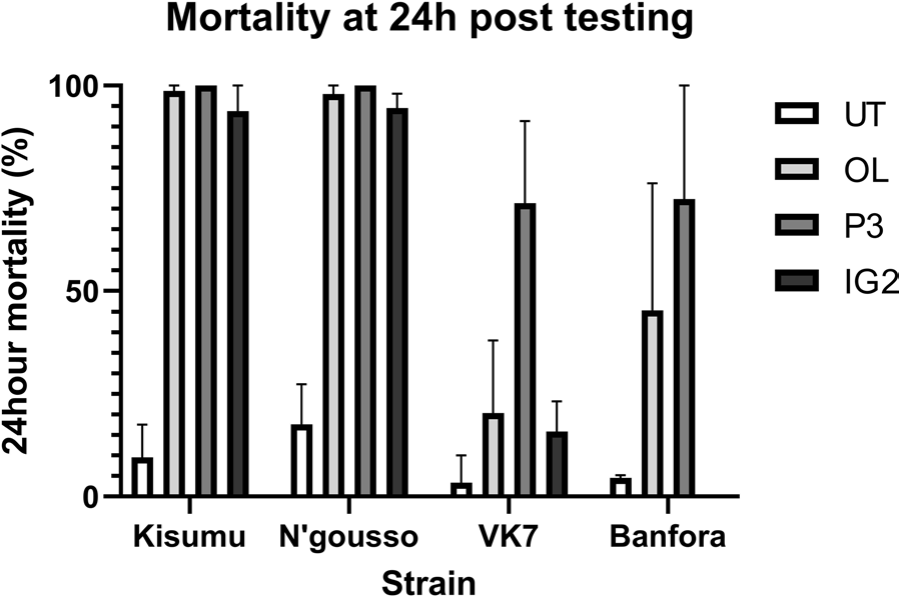
Mean mortality of two susceptible (Kisumu, and N’gousso) and two resistant (VK7 and Banfora) *Anopheles gambiae* strains at 24 h after a 2-h exposure during room scale tracking to untreated net (UT), Olyset Net (OL), PermaNet 3.0 (P3) and Interceptor G2 (IG2) with 95% Confidence Intervals

Mortality at 24 h after the 2-h room scale tracking assay on untreated net (UT) was below 20% for all strains (Fig. 2). OL net, P3 net and IG2 net all killed more than 90% of susceptible strains within 24 h. Mortality rates at 24 h were significantly lower for resistant VK7 and Banfora strains with OL, P3 and IG2 nets (Fig. 2) (Additional file 1: Table S1) compared to susceptible strains Kisumu and N’gousso (OL net: VK7 v Kisumu p < 0.0001, VK7 v N’gousso p < 0.0001, Banfora v Kisumu p = 0.0013, Banfora v N’gousso p = 0.0014; P3 net: VK7 v Kisumu p = 0.0042, N’gousso v VK7 p = 0.0903, N’gousso v Banfora p = 0.0602 Banfora v Kisumu p = 0.0007; IG2 net: VK7 v Kisumu p < 0.0001, VK7 v N’gousso p < 0.0001) (Additional file 1: Table S2). Note that the N’gousso results derive from only 2 test repeats, which may account for the non- significant *P-*values, despite the differences in mean mortalities. The highest 24 h mortality observed for VK7 strain was following P3 net tests, which was significantly higher than that of OL net (p = 0.0009) and IG2 net (p < 0.0001). There was no significant difference in mortality rates between OL net and IG2 net. Twenty-four-hour mortalities of the Banfora mosquitoes were 45.34% on OL net and 72.38% on P3 net and were not significantly different between ITNs.

Cumulative mortality rates 72 h after exposure to IG2 net (containing the slower acting pyrrole insecticide chlorfenapyr) were lower in VK7 than in both susceptible strains (VK7 25.25%, 95% CI 10.29, 40.21]; Kisumu 95.91%, 95% CI [86.91, 100]; N’gousso 98.86%, 95% CI [95.25, 100]; VK7 v Kisumu t(8) = 9.28, p < 0.0001; VK7 v N’gousso t(8) = 10.04, p < 0.0001). Cumulative 72 h mortality for VK7 and Banfora after exposure to OL net increased to 35.04% and 61.42% respectively, and after P3 net exposure to 79.29% and 73.53%, respectively. The increase in mortality between 24 and 72 h seen after all ITN exposure was not significantly different than the increase seen in this time frame after exposure to UT nets for either resistant strain.

#### Longevity

**Fig. 3:**
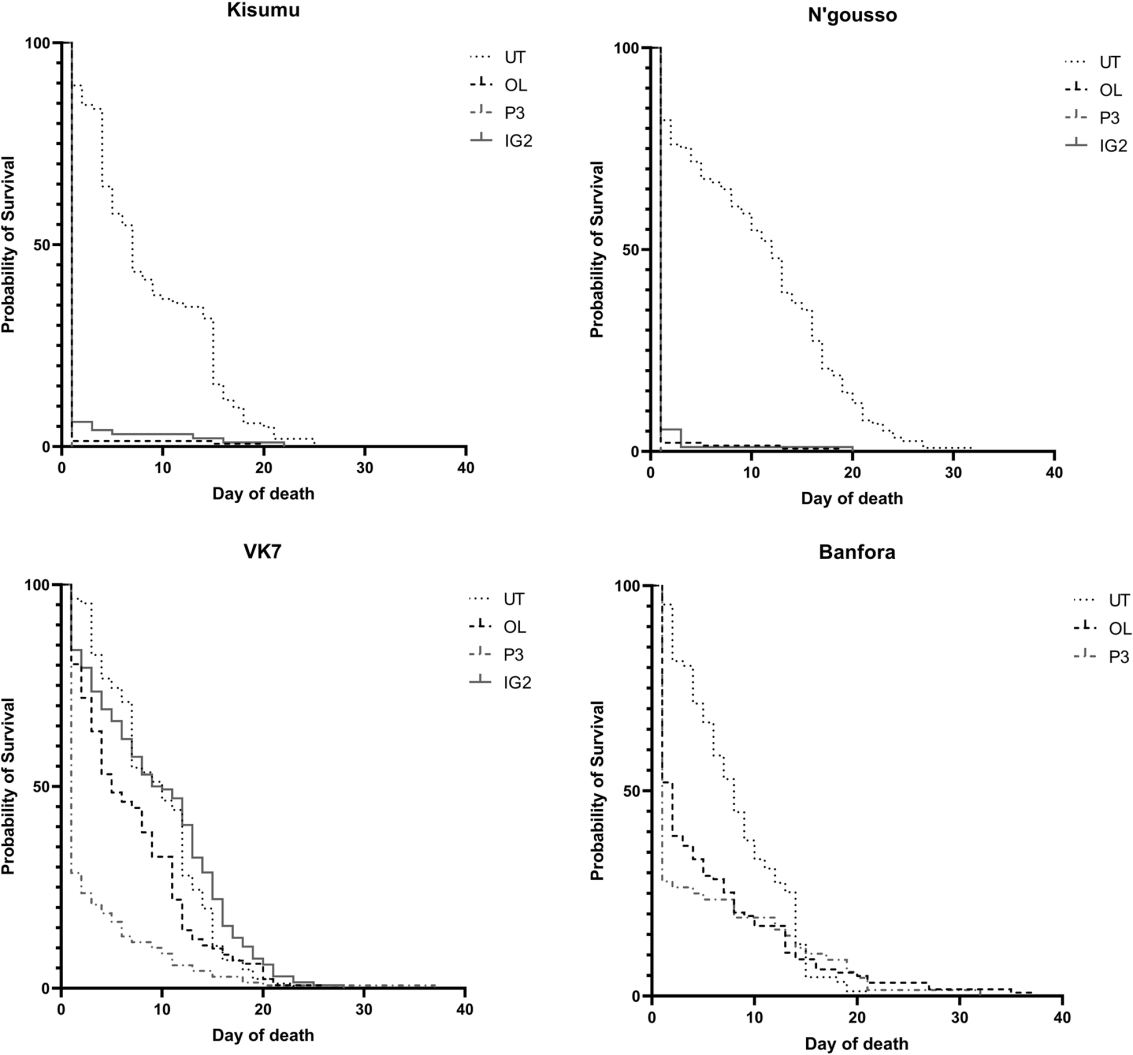
Survival curves for susceptible (Kisumu and N’gousso) and resistant (VK7 and Banfora) *Anopheles gambia*e after exposure in the room scale tracking room to either untreated net (UT), Olyset Net (OL), PermaNet 3.0 (P3) or Interceptor G2 (IG2). Day 0 is day of exposure

For VK7, median survival time after IG2 net exposure was not significantly different to that recorded after UT net exposure [IG2 net 10 days [95% CI 7.53, 12.48]; UT net 10 days [95% CI 8.23, 11.77]] with no significant difference in overall longevity [VK7 UT net v IG2 net p = 0.2150]. For the same strain, median survival times following OL net exposure was five days [95% CI 3.20, 6.80] and following P3 net was one day [95% CI 0, 1]. In both resistant strains, P3 net exposure had the largest impact in reducing longevity (VK7: UT net v OL net p = 0.0198, UT net v P3 net p < 0.0001; Banfora: UT net v OL net p = 0.0026, UT net v P3 net p = 0.0099) (Fig. 3). Both resistant strains survived significantly longer after exposure to all three ITNs compared to the susceptible strains (Additional file 1: Table S3). The median survival time after exposure to UT nets varied between strains (Kisumu 7 days [95% CI 5.58, 8.33]; N’gousso 12 days [95% CI 10.25, 13.76]; VK7 10 days [95% CI 8.23, 11.77]; Banfora 8 days [95% CI 6.49, 9,51]).

### Mosquito activity and behaviour

#### Total activity and behavioural mode

**Fig. 4:**
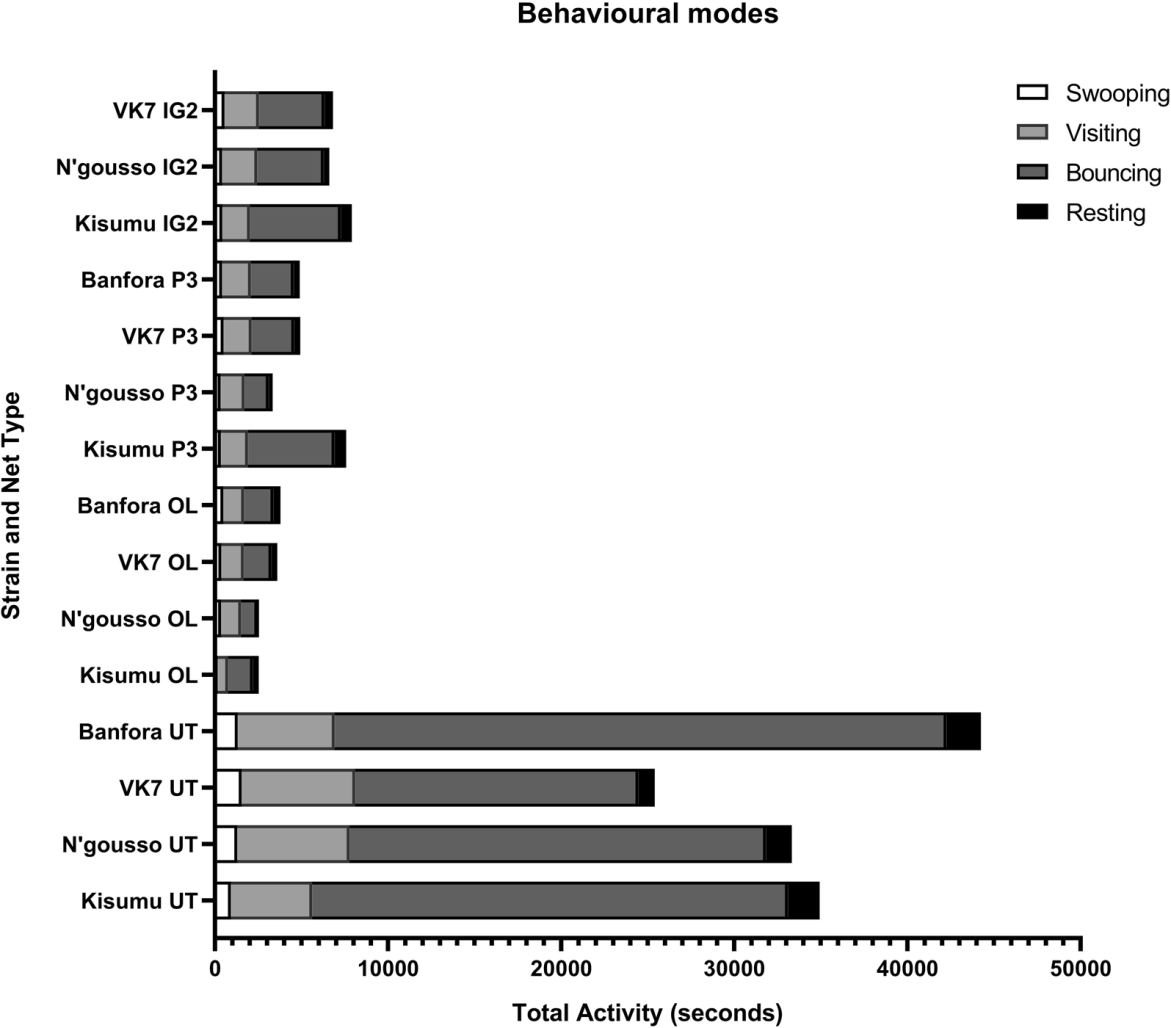
Behaviour of *Anopheles gambiae* at human baited bed nets. Mean total activity time of *An. gambiae* recorded for each behavioural mode over 2-h recording period. As multiple mosquitoes were active simultaneously in the field of view, the total activity time could exceed the total recording time of 2 h (7200 s)

Figure 4 shows mean total mosquito activity for each strain and net combination, across a 2-h recording, with activity separated into the four distinct behavioural modes: swooping, visiting, bouncing or resting as defined by Parker et al. [16]. Across all treatments, flight track length ranged from 2.5 mm to 20,249 mm and track duration ranged from 0.08 s to 1,010 s. For all four strains, total activity was significantly longer at an UT net than at any of the three ITNs (Kisumu Welch’s F(3.0, 8.71) = 44.44, p < 0.0001; N’gousso Welch’s F(3.0, 3.59) = 24.15, p = 0.0074; VK7 Welch’s F(3.0, 7.27) = 20.82, p = 0.0006; Banfora Welch’s F(2.0, 5.29) = 32.17, p = 0.0011). Comparing net types showed no significant differences in total activity between any of the strains (UT net Welch’s F(3.0, 6.90) = 3.94, p = 0.0626; OL net Welch’s F(3.0, 9.38) = 2.21, p = 0.1543; P3 net Welch’s F(3.0, 4.11) = 2.23, p = 0.2240; IG2 net Welch’s F(2.0, 9.30) = 0.60, p = 0.5709).

Breaking down total mosquito activity to look at time spent in each of the four distinct behavioural modes, revealed that both susceptible and resistant mosquitoes always spent more time swooping, visiting, bouncing and resting at an UT net than at any of the three ITNs (Additional file 1: Table S4; the one exception to this was comparing VK7 on UT net and IG2 net, where there was no difference in total time spent resting (VK7 UT net v IG2 net p = 0.1591)). However, there were no significant differences in the proportionate amounts of time spent swooping, visiting, bouncing, or resting between different ITNs (Additional file 1: Table S5).

Results comparing total activity changes on each net between strains for the four behavioural modes, showed that there was no difference in swooping activity between any strains on any nets, bar VK7 showing more activity than Kisumu around an UT net (UT net Kisumu v VK7 p = 0.0010). Analysis of total visiting time showed that N’gousso and VK7 spent more time in this behavioural mode than Kisumu when an UT net was present (UT net Kisumu v N’gousso p = 0.0352, Kisumu v VK7 p = 0.0248), but there were no differences when comparing between any other nets. Banfora spent significantly more time bouncing on UT net than all other strains (UT net Kisumu v Banfora p = 0.0014, N’gousso v Banfora p < 0.0001, VK7 v Banfora p < 0.0001), and both susceptible strains spent more time bouncing than resistant VK7 (Kisumu v VK7 p < 0.0001 N’gousso v VK7 p = 0.0032). There was no difference in time spent bouncing between any strains on any of the ITNs. Kisumu and Banfora spent more time resting on an UT net than VK7 (UT net Kisumu v VK7 p = 0.0004, VK7 v Banfora p = 0.0001), but there were no other significant differences in total time spent resting with an UT net or any of the ITNs (Additional file 1: Table S6).

### Quantifying number and duration of net contact

#### Contact number

**Fig. 5:**
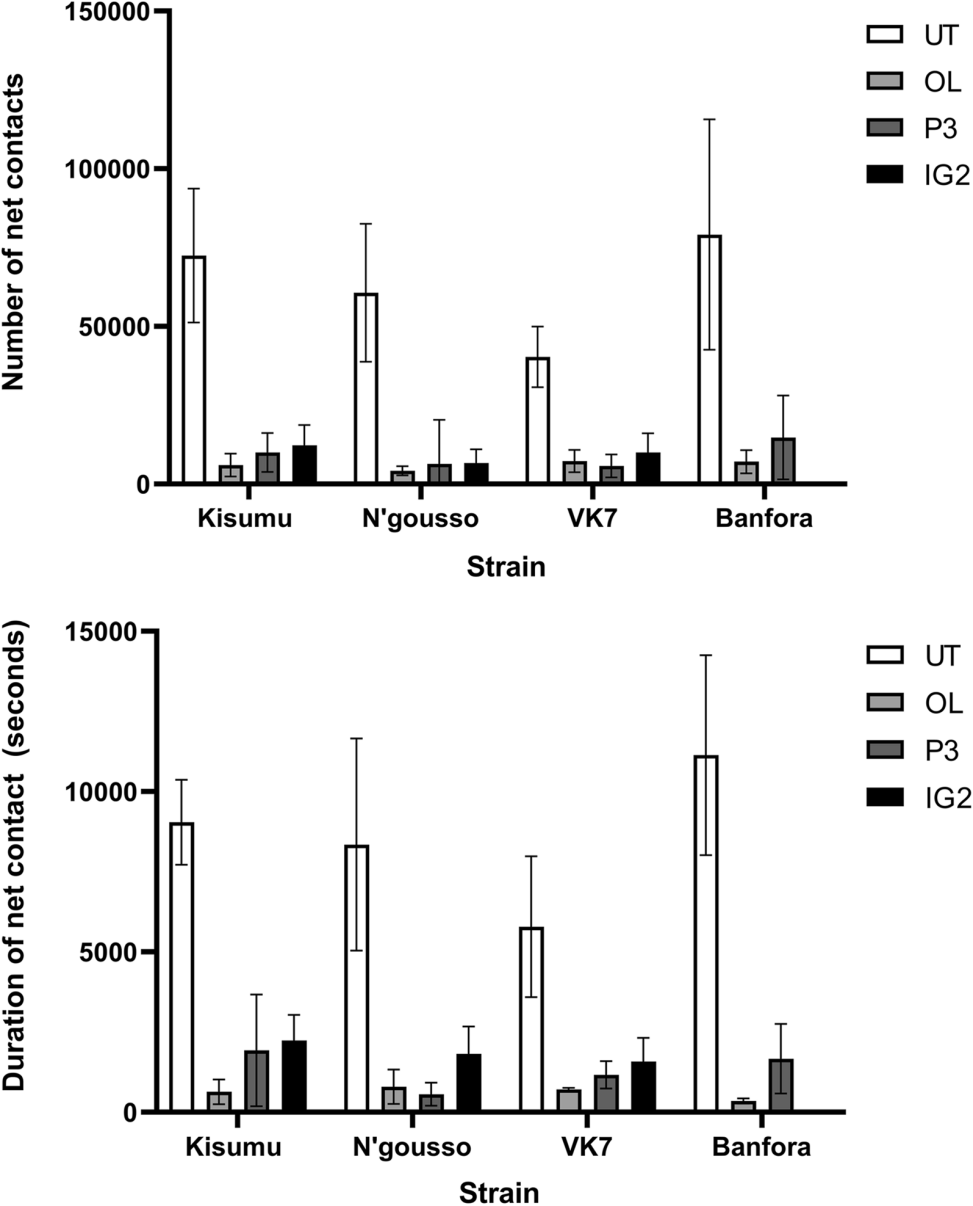
Mean total number of net contacts and mean total duration of net contact with 95% Confidence Intervals for susceptible (Kisumu and N’gousso) and resistant (VK7 and Banfora) Anopheles gambiae strains on untreated net (UT), Olyset Net (OL), PermaNet 3.0 (P3) and Interceptor G2 (IG2)

All strains showed significantly greater mean total number of contacts with the UT net than with any of the ITNs (Additional file 1: Table S7). There were significant differences in the mean number of contacts with an UT net between some strains: Banfora had significantly more contact with the UT net than N’gousso and VK7, while Kisumu and N’gousso had more contact than VK7. Within strain comparisons showed there was no significant difference in the number of contacts made with any of the ITNs (Additional file 1: Table S8). There was also no difference in the number of contacts made between any of the strains on any of the ITNs (Additional file 1: Table S9) (Fig. 5, panel A).

#### Contact duration

Both susceptible and resistant mosquitoes spent significantly more time in contact with the UT net than any of the ITNs. Kisumu spent significantly more time in contact with IG2 net than OL net, but there were no other differences between nets (Additional file 1: Table S10). Between strain comparisons showed that Banfora spent significantly more time on UT net than all other strains, and both susceptible strains had longer contact duration than VK7. There was no significant difference in net contact duration for any strain combinations on treated nets (Additional file 1: Table S11) (Fig. 5, panel B).

It was calculated that during the 120-min recording period each mosquito had between 285.62 s and 1041.79 s of contact with the UT net. There were no significant differences in the minimum and maximum time that susceptible and resistant mosquitoes spent on any of the three ITNs (OL net: susceptible strains between 7.58 s and 101.39 s, resistant strains between 3.39 s and 255.53 s; P3 net: susceptible strains between 40.30 s to 241.77 s, resistant strains 33.35 s to 273.47 s; IG2 net: susceptible strains between 40.45 s and 403.39 s, resistant strain between 34.44 s and 378.73 s). The only notable differences observed were that the minimum time that one Kisumu mosquito could have spent on OL net was significantly lower than IG2 net (p = 0.0344), and the maximum time that N’gousso spent on IG2 net was longer than on OL net (p = 0.0243) (Table 4).

**Table 4:** Minimum and maximum individual mosquito net contact duration (seconds) for entire 120 min recording

Treatment	Strain	Minimum contact duration (s)	Maximum contact duration (s)
Untreated	Kisumu	301.45	952.28
	N’gousso	398.72	962.83
	*VK7*	285.62	714.06
	*Banfora*	542.17	1041.79
Olyset Net	Kisumu	7.58	101.39
	N’gousso	9.96	64.28
	*VK7*	18.7	77.93
	*Banfora*	3.39	255.53
PermaNet 3.0	Kisumu	40.3	241.77
	*VK7*	33.35	273.47
	*Banfora*	46.65	323.24
Interceptor G2	Kisumu	52.44	403.39
	N’gousso	40.45	341.07
	*VK7*	34.44	378.73

Resistant mosquito strains are denoted in italics

### Net interactions in first 10 min of assay

Net contact was investigated in the first 10 min of the video tracking to examine if there was any indication of immediate repellent effects of the ITNs. While contact number and contact duration were lower at ITNs than UT nets, a higher percentage of overall contact duration occurred in the first 10 min of the assay on ITNs for the susceptible strains (Table 5). In the first 10 min, Kisumu spent significantly more time in contact with the ITNs than UT, and more time in contact with IG2 net than OL net or P3 net. Similarly, N’gousso had a higher percentage of contact time occurring in the first part of the assays when OL net and IG2 net were present, compared to the UT net. Again, N’gousso also had a longer contact duration on IG2 net than OL net. For resistant VK7, the highest initial 10-min contact duration was observed on P3 net, whereas Banfora showed similar time spent across all three treatments.

**Table 5:** Percentage of overall contact duration occurring in the first 10 min of the 2 h assay [95% confidence intervals]

Net	Strain	% 10 min [95% CI]
Untreated net	Kisumu	5.49 [3.43, 7.55]
	N’gousso	8.58 [− 0.26, 17.42]
	*VK7*	1.81 [0.16, 3.46]
	*Banfora*	4.65 [1.91, 7.40]
Olyset Net	Kisumu	48.13 [21.76, 74.50]
	N’gousso	55.86 [38.31, 73.41]
	*VK7*	1.27 [− 1.93, 4.47]
	*Banfora*	6.39 [− 1.09, 13.87]
PermaNet 3.0	Kisumu	29.68 [10.70, 48.65]
	N’gousso	31.73 [− 59.07, 122.53]
	*VK7*	23.73 [5.20, 42.26]
	*Banfora*	11.75 [3.64, 19.85]
Interceptor G2	Kisumu	38.57 [33.29. 43.85]
	N’gousso	34.67 [17.65, 51.68]
	*VK7*	6.00 [1.01, 10.98]

Resistant mosquito strains are denoted in italics

Despite differences within strains on different nets, there were no differences observed between susceptible and resistant strains for 10-min contact duration when an UT net or P3 net was present. There was, however, a difference with OL net, as both susceptible strains had a higher percentage of their overall contact duration occurring in this first period than both resistant strains. Susceptible strains also spent considerably more time contacting IG2 net than VK7 (Additional file 1: Tables S12 S13, S14, S15).

### Location of activity at the bed net interface

The distribution of total activity was heavily focused on the roof of the bed net for all strains and all net treatments (> 90% on UT net, > 85% OL net, > 72% P3 net and > 87% IG2 net) as described in previous studies on standard ITNS [17, 29] (Table 6). There was no significant difference in the percentage of contact occurring on the roof of the net for any strain or net combinations.

**Table 6:** Percentage of overall contact across different regions of the bed net (%)

Treatment	Strain	Roof	Front	Sides
Untreated	Kisumu	93.91	5.81	0.28
	N’gousso	96.49	2.83	0.69
	*VK7*	91.64	7.51	0.86
	*Banfora*	95.73	3.47	0.80
Olyset Net	Kisumu	92.58	7.09	0.33
	N’gousso	86.39	10.27	3.34
	*VK7*	86.59	11.99	1.42
	*Banfora*	85.22	13.15	1.63
PermaNet 3.0	Kisumu	72.19	25.66	2.15
	*VK7*	78.67	16.22	5.11
	*Banfora*	91.61	5.84	2.55
Interceptor G2	Kisumu	92.33	6.64	1.03
	N’gousso	92.23	6.53	1.24
	*VK7*	87.87	9.77	2.36

Resistant mosquito strains are denoted in italics

### Mosquito velocity during interaction with host within bed nets

The average speed of whole swooping tracks was analysed to assess changes in speed between strains around different bed nets. Only susceptible Kisumu showed any difference in flight speed around different net treatments, flying significantly faster around OL nets and IG2 nets than UT nets. Resistant strains did now show any difference in flight speed between different net types. Between strains, both resistant strains flew faster around an UT net than Kisumu and Banfora was significantly faster than Kisumu around P3 nets. There was no difference in overall swooping speed between strains when OL net or IG2 net were present (Additional file 1: Tables S16, S17).

### Mosquito interaction with the bed nets over time

**Fig. 6:**
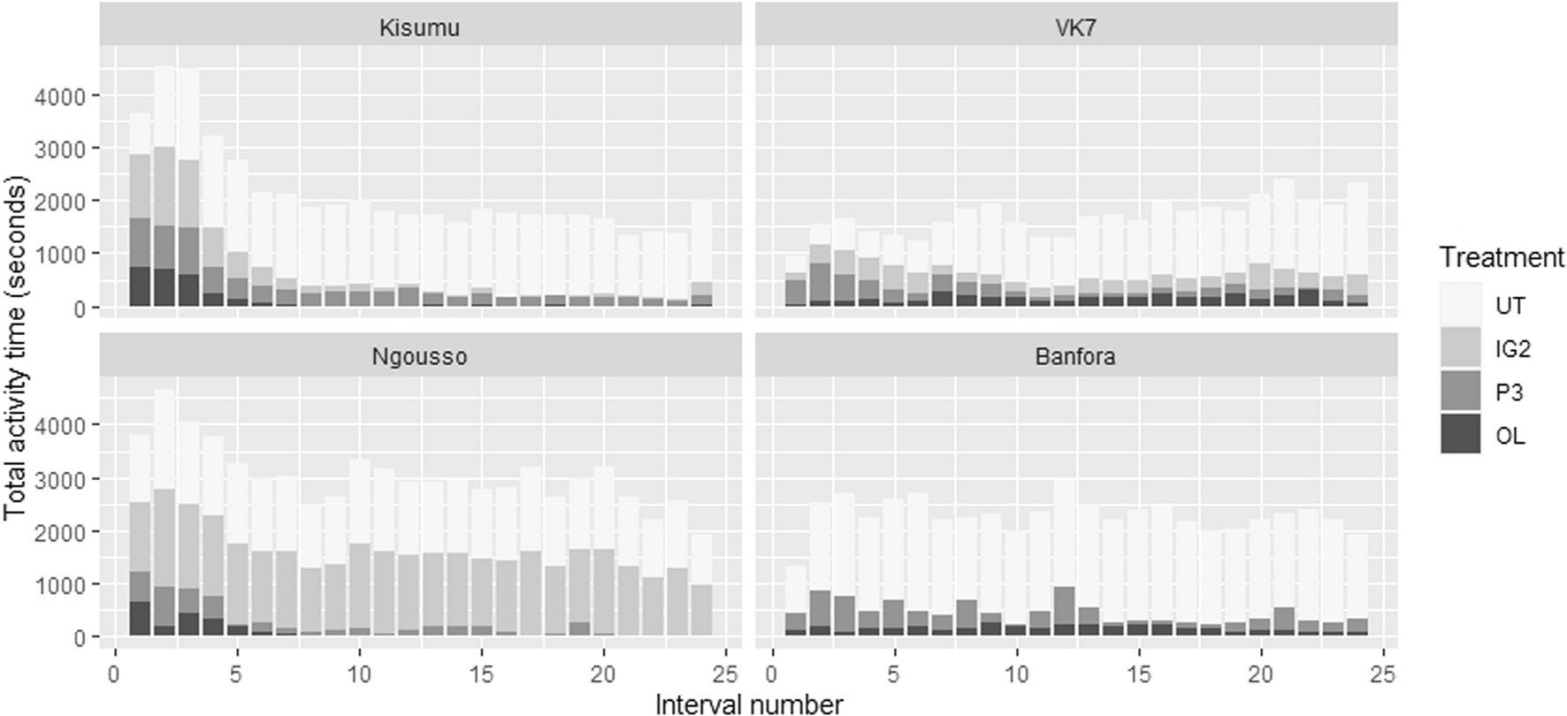
Rates of mosquito activity across all four behavioural modes combined for entire 120-minute recording test period. Total activity is shown for untreated net (UT), Olyset Net (OL), PermaNet 3.0 (P3) and Interceptor G2 (IG2) for Kisumu, N’gousso, VK7 and Banfora

A steep decay in activity over the duration of the assay was observed for susceptible strains with P3 net and OL net compared to UT net (Kisumu: UT net v OL net p = 0.0023, UT net v P3 net p = 0.0020). Kisumu also showed a dramatic decrease in activity in the presence of IG2 net (UT net v IG2 net p < 0.0001), which was not replicated in N’gousso activity decay around the same net. Resistant strains showed a less extreme decay in activity when P3 net and OL net were present, however decay was still more pronounced than with UT net (VK7 UT net v OL net p = 0.0128, UT net v P3 net p = 0.0010), and there was no significant activity decay when VK7 was exposed to IG2 net. All strains exhibited no activity decay in the presence of an UT net (Fig. 6) (Additional file 1: Tables S18, S19).

### Sub-lethal pipeline—wing size and willingness to feed

Wing size was measured as it is a widely used proxy for mosquito body size [, 30,31]. There was a negative correlation between wing size and blood-feeding inhibition, with smaller mosquitoes less likely to survive and accept a blood meal. However, there was no significant interaction between wing size and strain (p = 0.9447), suggesting that the relationship between wing size and blood feeding success was similar for all strains.

**Fig. 7:**
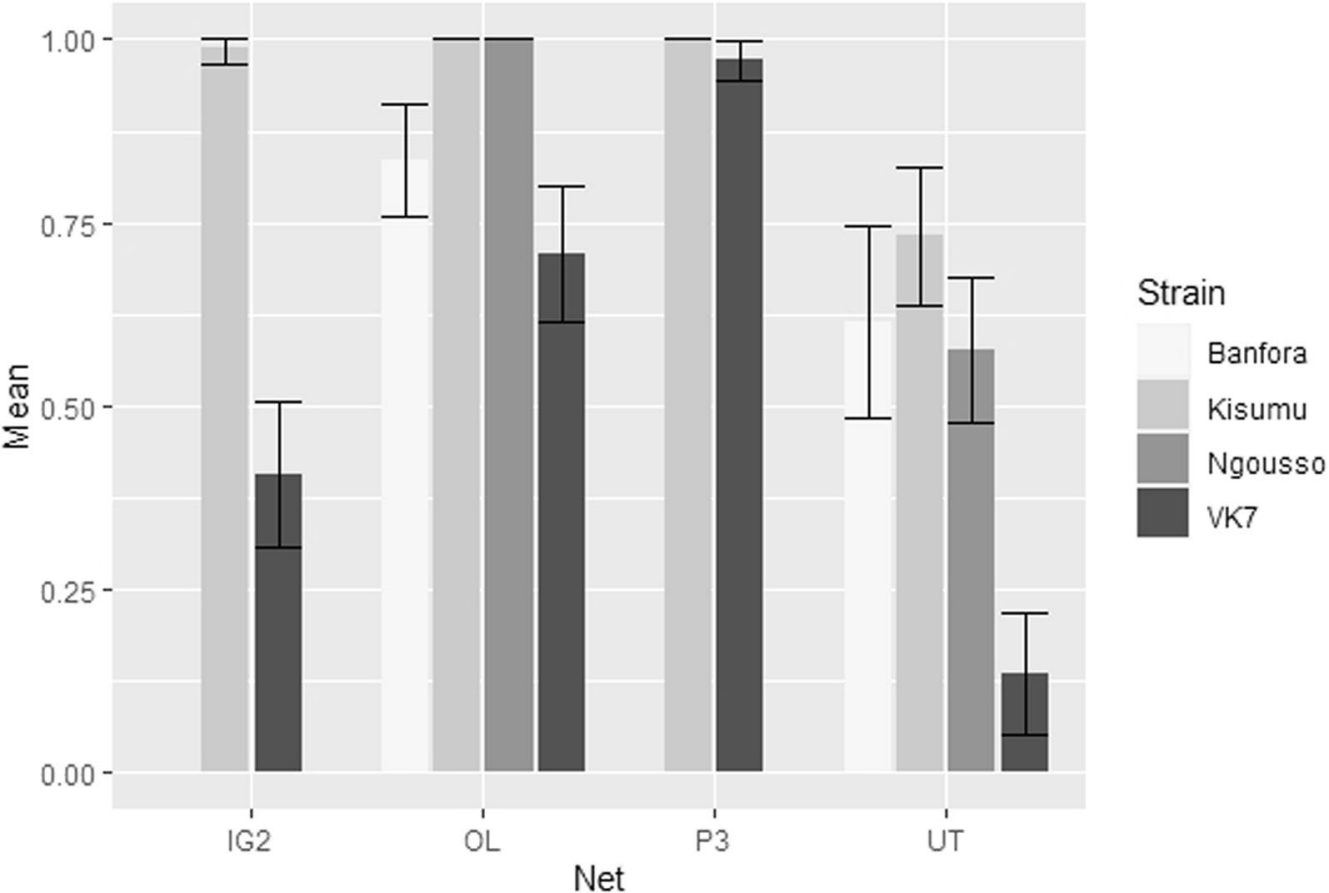
Mean reduction in blood feeding success for all four mosquito strains after exposure to Interceptor G2 (IG2), Olyset Net (OL), PermaNet 3.0 (P3) and Untreated Net (UT)

The majority of susceptible mosquitoes exposed to the three ITNs were either knocked-down or dead and hence unable to blood feed. OL net reduced resistant strain feeding by up to 83% (VK7 71% [95% CI 62, 80], Banfora 83% [95% CI, 76, 91]), P3 net reduced VK7 feeding by 97% [95% CI 94, 99], whereas IG2 net had a lesser effect, reducing VK7 blood feeding success by 41% [95% CI 31, 51] (Fig. 7). Between 14 and 70% of mosquitoes were unable to blood feed after exposure to UT net.

## Discussion

These results provide a first in-depth description of the behaviour of susceptible and resistant *An. gambiae* strain mosquitoes at next-generation bed nets and the impact of these new nets on them. These data come at a critical time for ITNs. As insecticide resistance continues to be a growing threat to the success of malaria vector control programmes, there is an urgent need for safe novel treatments suitable for use with ITNs. The first of the next-generation nets using these treatments have been evaluated in field trials [7, 8] and are deployed at scale in pilot studies in several countries [32]. Determining how mosquitoes interact with these new nets, and the consequences of net contact for mosquitoes, will aid in interpretation of the results of clinical trials, and extrapolation to alternative settings with different mosquito populations.

The results indicate (Fig. 3) that the new nets (P3 and IG2) all killed more than 90% of susceptible mosquitoes 24 h after a 2-h exposure, but had a lesser impact on resistant strains,with P3 killing only 71.4% of VK7 and 72.4% of Banfora, and IG2 killing 15.9% of VK7 (the Banfora strain was not tested on this net). The pyrethroid only OL nets performed similarly to the dual treatment nets, killing 45% of resistant mosquitoes. Total mosquito activity was higher around a UT net than all ITNs, which is comparable with results obtained in previous studies [16, 17]. Interestingly, there was no difference in total activity observed between susceptible and resistant strains around any of the ITNs tested, the number and duration times of net contact was also similar for all strains. Net contact was focussed predominantly on the roof for all types of bed net and did not change throughout the assay [16, 31]. When comparing the difference in the first and last 10 min of recording activity (Table 5), there was a steep decay in activity for both susceptible strains when P3 net and OL net were present, but only a decrease in activity around IG2 net for susceptible Kisumu. Resistant strains showed a less steep decay in activity when P3 net and OL net were present, though the decay was still more pronounced than with UT. The steeper activity decay in susceptible strains most likely indicates that mosquitoes were being knocked down rapidly and killed by the active-ingredients, but the absence of decay with the resistant strains was surprising, particularly in assays with dual-treated nets, indicating that the second AI is not immediately impacting the flight of these resistant mosquitoes.

The behaviour of the various mosquito strains, as measured by tracking, was remarkably consistent across all those tested, with no significant differences observed in the number of contacts, or the duration of time spent in contact with the ITNs between both susceptible and resistant mosquito strains. No evidence of a repellent effect on susceptible mosquitoes was observed for any ITN, as a higher percentage of overall contact duration occurred during the first 10 min of the assay on all ITNs compared to untreated nets. Moreover, for every combination of mosquito strain and insecticide tested, the most visited bed net location was the roof, that repellent effects at the net were minimal or negligible, and that the durations of net contact at the new dual AI nets were not dissimilar to those previously described for the Permanet 2 standard net. Hence despite the differences in the modes of action of the AIs used on the various ITNs tested here, the entomological modes of action seem remarkably similar, and there is no indication from the behaviour recorded here that the Dual AI nets will perform any differently to the existing standard nets in practice.

The low mortality results in resistant strains from our study do not match those from recent experimental hut studies reporting promising results with the Interceptor G2 net [33-35, 35] where mortality in huts with IG2 net was significantly higher than with standard pyrethroid only ITNs in all settings. Recent clinical trials reported that after two years, IG2 net provided significantly better protection from malaria than an alpha-cypermethrin only ITN in areas where mosquito populations are resistant to pyrethroids [7, 8]. Nevertheless, when tested in a laboratory under standard conditions, the results from ours and other studies are not dissimilar, with low mortalities of insecticide resistant mosquitoes at both 24 h and 72 h post IG2 net exposure. 25.6% mortality at 72 h in the resistant VK7 strains was recorded during this study, and low mortalities have also been recorded in other laboratory bioassays using IG2 [34, 35]. The reasons for differences in performance of IG2 net under laboratory and field settings are unclear but differences in the mosquito population assessed may be important. Mosquito strains that have been maintained under insectary conditions for many years could behave differently to wild mosquitoes due to their clean, closed, constant environments and regular feeding regimes. Unpublished data from multiple experimental hut studies in southwest Burkina Faso (the region of origin of the VK7 and Banfora strains used in the current study) show relatively poor performance of IG2 nets compared to data from other settings (Sanou, A, Sagnon N, Guelbeogo M).

Moreover, chlorfenapyr has a complex mode of action; the pro-insecticide is metabolized to its active form in the mosquito which then disrupts energy production in the mitochondria. Understanding how the Absorption, Distribution, Metabolism and Excretion (ADME) of chlorfenapyr differ between mosquito populations with differing pyrethroid resistance mechanisms, and how the behaviour of the mosquito around ITNS may impact the mode of action, is critical to develop and apply appropriate lab tests, to evaluate products containing this insecticide.

Mosquitoes were given the opportunity to blood feed 1-h post-assay, where it was observed that there was a reduction in blood feeding success with resistant strains after exposure to all ITNs. Despite low levels of mortality with the pyrethroid only Olyset Net and next-generation Interceptor G2, blood feeding success in resistant strains was reduced by up to 83% and 41% respectively. A reduction in blood-feeding following insecticide exposure was also found by Barreaux et al. [36] who reported that after forced exposure to ITNs the blood feeding success of highly insecticide resistant *An. gambiae* strains was reduced. The authors suggest that this was not a result of mosquitoes avoiding the net or being repelled by it, but instead because contact with insecticides reduced feeding capacity.

As previously observed [17], both susceptible and resistant strains showed a much higher level of host-seeking activity at a UT net, with markedly lower activity levels in the presence of all tested ITNs. This reduction in activity was observed for all strains with no significant differences in total activity level between any of the strain and ITN comparisons. This suggests that the novel chemistries do not affect the behaviour of mosquitoes of differing resistance status differently. One result to note, is that despite the low mortality rate of VK7 when exposed to IG2 net, the time spent resting on this ITN was similar to that of when an UT net was present. It is not clear why such prolonged net contact resulted in such low levels of mortality in this, a resistant strain and the experiments need repeating with additional resistant strains before any assumptions are made.

There are several limitations to this study, which are important to consider. While the environment in which the tracking assay data are collected reproduces as much as possible the conditions in the interior of a hut, there are important omissions and differences. Firstly, any repellent properties that may reduce initial eave entry cannot be measured here nor can the proportion of mosquitoes that leave the room after contacting the net. Hence all 25 mosquitoes must enter and remain in the room potentially delivering an overestimate of the lethality of the net being tested. Environmental conditions also remained static throughout the test whilst in reality air disturbances, and changes in temperature during the night may affect net contact.

It was not possible to determine individual mosquito contact with this tracking system, and total net contact was calculated based on the maximum number of mosquitoes seen simultaneously contacting the net in any one frame of the recording. Although this method provides a more realistic estimate of mosquito/ITN contact times than other standard bioassays, the measurement does not account for mosquitoes that make zero contact or that return to make multiple contacts with the net. This is especially important for the interpretation of sublethal results with contact duration varying between the individuals exposed. There is, therefore, a strong argument for collecting data to determine LD50 equivalents for duration of net contact, determined for each ITN. The video recordings in this study were limited to 2 h as the data files produced are extremely large (2–3 Tb per camera, per recording), but recording mosquito behaviour for longer periods to assess any delayed effects on mosquito behaviour could prove important when evaluating impacts of nets with poorly understood AIs. Future studies would benefit from more replicates with multiple different resistant mosquito strains, to investigate the potential effect of different resistance mechanisms, as previously advocated [37].

Overall, these findings expand our knowledge of how mosquitoes interact with ITNs, particularly with regards to the impact of the new chemistries on the vector. These results indicate that the effects of a range of ITNs on mosquito behaviour is remarkably consistent with no major alterations in mosquito responses by mosquitoes with different pyrethroid susceptibilities. It is also clear that reduced ITN contact is not the reason for observed lower mortality in resistant strains. Ongoing work in multiple field sites will continue to explore the effects of new ITNs on the behaviour of wild mosquito populations and may provide new insights into the entomological mode of action of next generation nets.

## Supplementary Information

**Additional file 1: Table S1**. Mean 24hour mortality [95% CI]. Resistant mosquito strains are denoted in italics. **Table S2**. Mean 24hour mortality comparisons between three insecticide treated nets and four mosquito strains, two susceptible (Kisumu and N’gousso) and two resistant (VK7 and Banfora). Resistant mosquito strains are denoted in italics. **Table S3**. Comparison of median survival times of susceptible (Kisumu and N’gousso) and resistant (VK7 and Banfora) strains on four different net treatments.  Resistant mosquito strains are denoted in italics. **Table S4**. Statistically significant differences (p values) in total activity time split into four different behavioural modes (swooping, visiting, bouncing and resting), comparing untreated (UT) net to either Olyset Net (OL), PermaNet 3.0 (P3) or Interceptor G2 (IG2), for susceptible (Kisumu and N’gousso) and resistant (VK7 and Banfora) mosquitoes. Resistant mosquito strains are denoted in italics. **Table S5**. Within strain comparisons (p-value) of total activity time split into four different behavioural modes (swooping, visiting, bouncing and resting) between three ITNs (Olyset Net = OL, PermaNet 3.0 = P3, Interceptor G2 = IG2). Resistant mosquito strains are denoted in italics. **Table S6**. Within treatment comparisons (p-value) of total activity split into four behavioural modes (swooping, visiting, bouncing and resting) on four ITNs (Untreated net = UT, Olyset Net = OL, PermaNet 3.0 = P3, Interceptor G2 = IG2) between four mosquito strains. Resistant mosquito strains are denoted in italics. **Table S7**. Mean total number of bed net contacts [95% CI], mean total contact duration [95% CI] and maximum number of mosquitoes seen in one frame of video recording. Resistant mosquito strains are denoted in italics. **Table S8**. Within strain statistical comparisons (p value) of total number of net contacts for susceptible (Kisumu and N’gousso) and resistant (VK7 and Banfora) mosquitoes between four nets (UT = untreated, OL = Olyset Net, P3 = PermaNet 3.0, IG2 = Interceptor G2). Resistant mosquito strains are denoted in italics. **Table S9**. Within treatment statistical comparisons (p value) of total number of net contacts for four nets between four mosquito strains. Resistant mosquito strains are denoted in italics. **Table S10**. Within strain comparisons (p-value) of total duration of net contact for susceptible (Kisumu and N’gousso) and resistant (VK7 and Banfora) mosquitoes between three ITNs (OL = Olyset Net, P3 = PermaNet 3.0, IG2 = Interceptor G2). Resistant mosquito strains are denoted in italics. **Table S11**. Within treatment comparison (p-value) of total net contact duration for three ITNs between four mosquito strains.  Resistant mosquito strains are denoted in italics. **Table S12**. Percentage of contact duration in first the 10 minutes of room scale tracking assay – within strain, between net differences. Resistant mosquito strains are denoted in italics. **Table S13**. Percentage of contact duration in first 10mins of assay – within net, between strain differences Resistant mosquito strains are denoted in italics. **Table S14**. Average contact duration in first 10 minutes—within strain, between net comparisons. Resistant mosquito strains are denoted in italics. **Table S15**. Average contact duration in first 10minutess – within net, between strain comparisons. Resistant mosquito strains are denoted in italics. **Table S16**. Comparison (p-value) of average swooping speeds across 2hour assay within four different strains, between four different net treatments. Resistant mosquito strains are denoted in italics. **Table S17**. Comparison of average swooping speeds across 2hour assay within four net treatments, between four strains. Resistant mosquito strains are denoted in italics. **Table S18**. Comparison of activity decay over time (p-value), within strain, between net treatment. Resistant mosquito strains are denoted in italics. **Table S19**. Comparison of activity decay over time (p-value), within net treatment, between strains. Resistant mosquito strains are denoted in italics.

## Data Availability

The data sets used for the work in this article are available upon request from the authors.

## Abbreviations

ADME: Absorption, distribution, metabolism, excretion
AI: Active ingredient
Fps: Frames per second
GLM: Generalized liner model
IG2 net: Interceptor G2 net
ITNs: Insecticide-treated nets
kdr: Knockdown resistance
LSTM: Liverpool School of Tropical Medicine
MoA: Mode of action
OL net: Olyset Net
PBO: Piperonyl butoxide
P3 net: PermaNet 3.0
RH: Relative humidity
UT net: Untreated net
vgsc: Voltage gated sodium channel
WHO: World Health Organization
