# Supplementary material for: Impacts of dual active-ingredient bed nets on the behavioural responses of pyrethroid resistant Anopheles gambiae determined by room-scale infrared video tracking
Source: Malar J. 2023 Apr 23;22:132. doi: 10.1186/s12936-023-04548-9 (PMC10122874; doi:10.1186/s12936-023-04548-9)
Supplement: Supplementary file 1 — Additional file 1: Table S1. Mean 24hour mortality [95% CI]. Resistant mosquito strains are denoted in italics. Table S2. Mean 24hour mortality comparisons between three insecticide treated nets and four mosquito strains, two susceptible (Kisumu and N’gousso) and two resistant (VK7 and Banfora). Resistant mosquito strains are denoted in italics. Table S3. Comparison of median survival times of susceptible (Kisumu and N’gousso) and resistant (VK7 and Banfora) strains on four different net treatments. Resistant mosquito strains are denoted in italics. Table S4. Statistically significant differences (p values) in total activity time split into four different behavioural modes (swooping, visiting, bouncing and resting), comparing untreated (UT) net to either Olyset Net (OL), PermaNet 3.0 (P3) or Interceptor G2 (IG2), for susceptible (Kisumu and N’gousso) and resistant (VK7 and Banfora) mosquitoes. Resistant mosquito strains are denoted in italics. Table S5. Within strain comparisons (p-value) of total activity time split into four different behavioural modes (swooping, visiting, bouncing and resting) between three ITNs (Olyset Net = OL, PermaNet 3.0 = P3, Interceptor G2 = IG2). Resistant mosquito strains are denoted in italics. Table S6. Within treatment comparisons (p-value) of total activity split into four behavioural modes (swooping, visiting, bouncing and resting) on four ITNs (Untreated net = UT, Olyset Net = OL, PermaNet 3.0 = P3, Interceptor G2 = IG2) between four mosquito strains. Resistant mosquito strains are denoted in italics. Table S7. Mean total number of bed net contacts [95% CI], mean total contact duration [95% CI] and maximum number of mosquitoes seen in one frame of video recording. Resistant mosquito strains are denoted in italics. Table S8. Within strain statistical comparisons (p value) of total number of net contacts for susceptible (Kisumu and N’gousso) and resistant (VK7 and Banfora) mosquitoes between four nets (UT = untreated, OL = Olyset Net [file 12936_2023_4548_MOESM1_ESM.doc]

**Additional material**

**Additional Table 1. Mean 24hour mortality [95% CI].** **Resistant mosquito strains are denoted in *italics*.**

| **Treatment** | **Strain** | **24hour mortality (%) [95% CI]** |
| --- | --- | --- |
| Untreated | Kisumu | 9.5 [1.47, 17,54] |
|  | N’gousso | 17.64 [7.87, 27.40] |
|  | *VK7* | 3.36 [0, 10.05] |
|  | *Banfora* | 4.52 [3.85, 5.20] |
| Olyset Net | Kisumu | 98.67 [95.24, 100] |
|  | N’gousso | 97.97 [94.44, 100] |
|  | *VK7* | 20.35 [2.09, 38.01] |
|  | *Banfora* | 45.34 [14.52, 76.17] |
| PermaNet 3.0 | Kisumu | 100 [100, 100] |
|  | N’gousso | 100 [100, 100] |
|  | *VK7* | 71.37 [51.39,91.36] |
|  | *Banfora* | 72.38 [41.13, 100] |
| Interceptor G2 | Kisumu | 93.88 [81.53, 100] |
|  | N’gousso | 94.56 [91.10, 98.02] |
|  | *VK7* | 15.90 [8.62, 23.21] |

**Additional Table 2. Mean 24hour mortality comparisons between three insecticide treated nets and four mosquito strains, two susceptible (Kisumu and N’gousso) and two resistant (VK7 and Banfora). Resistant mosquito strains are denoted in *italics*.**

| **Strain comparison** | **ITN** | | |
| --- | --- | --- | --- |
|  | **Olyset Net** | **PermaNet 3.0** | **Interceptor G2** |
| Kisumu v *VK7* | t(9)= 12.80, p<0.0001 | t(10)= 3.68, p=0.0042 | t(8)= 16.64, p<0.0001 |
| Kisumu v *Banfora* | t(10)= 4.42, p0.0013 | t(7)= 5.81, p=0.0007 | N/A |
| N’gousso v *VK7* | t(9)= 12.67, p<0.0001 | t(6)= 2.07, p=0.0903 | t(8)= 21.54, p<0.0001 |
| N’gousso v *Banfora* | t(10)= 4.36, p=0.0014 | t(3)= 2.95, p=0.0602 | N/A |

**Additional Table 3. Comparison of median survival times of susceptible (Kisumu and N’gousso) and resistant (VK7 and Banfora) strains on four different net treatments.**  **Resistant mosquito strains are denoted in *italics*.**

| **Strain comparison** | **ITN** | | | |
| --- | --- | --- | --- | --- |
|  | **Untreated net** | **Olyset Net** | **PermaNet 3.0** | **Interceptor G2** |
| **Kisumu v N’gousso** | χ² (1, N=221) = 6.68, p=0.0098 | χ² (1, N=285) = 0.12, p=0.7241 | χ² (1, N=188) = 0.00, p>0.9999 | χ² (1, N=190) = 0.42, p=0.5191 |
| **Kisumu v *VK7*** | χ² (1, N=192) = 0.01, p=0.9733 | χ² (1, N=267) = 134.40, p<0.0001 | χ² (1, N=284) = 47.72, p<0.0001 | χ² (1, N=234) = 102.80, p<0.0001 |
| **Kisumu v *Banfora*** | Χ² (1, N=191) = 2.55, p=0.1102 | χ² (1, N=268) = 67.23, p<0.0001 | χ² (1, N=212) = 43.99, p<0.0001 | N/A |
| **N’gousso v *VK7*** | χ² (1, N=205) = 7.11, p=0.0077 | χ² (1, N=272) = 133.50, p<0.0001 | χ² (1, N=184) = 15.98, p<0.0001 | χ² (1, N=228) = 124.10, p<0.0001 |
| **N’gousso v *Banfora*** | χ² (1, N=204) = 15.67, p<0.0001 | χ² (1, N=263) = 65.16, p<0.0001 | χ² (1, N=112) = 14.67, p=0.0001 | N/A |
| ***VK7* v *Banfora*** | χ² (1, N=175) = 3.12, p=0.0773 | χ² (1, N=255) = 3.70, p=0.0545 | χ² (1, N=208) = 3.63, p=0.0568 | N/A |

**Additional Table 4. Statistically significant differences (p values) in total activity time split into four different behavioural modes (swooping, visiting, bouncing and resting), comparing untreated (UT) net to either Olyset Net (OL), PermaNet 3.0 (P3) or Interceptor G2 (IG2), for susceptible (Kisumu and N’gousso) and resistant (VK7 and Banfora) mosquitoes.** **Resistant mosquito strains are denoted in italics.**

| **Strain** | **Behaviour** | **Insecticide treated net** | | |
| --- | --- | --- | --- | --- |
|  |  | **Olyset Net** | **PermaNet 3.0** | **Interceptor G2** |
| Kisumu | swooping | <0.0001 | 0.0006 | 0.0067 |
|  | visiting | <0.0001 | <0.0001 | <0.0001 |
|  | bouncing | <0.0001 | <0.0001 | <0.0001 |
|  | resting | <0.0001 | <0.0001 | <0.0001 |
| N’gousso | swooping | <0.0001 | N/A | <0.0001 |
|  | visiting | <0.0001 | N/A | <0.0001 |
|  | bouncing | <0.0001 | N/A | <0.0001 |
|  | resting | <0.0001 | N/A | <0.0001 |
| *VK7* | swooping | <0.0001 | <0.0001 | <0.0001 |
|  | visiting | <0.0001 | <0.0001 | <0.0001 |
|  | bouncing | <0.0001 | <0.0001 | <0.0001 |
|  | resting | 0.0300 | 0.0264 | 0.1591 |
| *Banfora* | swooping | <0.0001 | <0.0001 | N/A |
|  | visiting | <0.0001 | <0.0001 | N/A |
|  | bouncing | <0.0001 | <0.0001 | N/A |
|  | resting | <0.0001 | <0.0001 | N/A |

**Additional Table 5. Within strain comparisons (p-value) of total activity time split into four different behavioural modes (swooping, visiting, bouncing and resting) between three ITNs (Olyset Net = OL, PermaNet 3.0 = P3, Interceptor G2 = IG2).** **Resistant mosquito strains are denoted in *italics*.**

| **Behaviour** | **ITN comparison** | **Strain** | | | |
| --- | --- | --- | --- | --- | --- |
|  |  | **Kisumu** | **N’gousso** | ***VK7*** | ***Banfora*** |
| Swooping | OL v P3 | 0.5855 | N/A | 0.8362 | 0.9811 |
|  | OL v IG2 | 0.1778 | 0.9800 | 0.5898 | N/A |
|  | P3 v IG2 | 0.8577 | N/A | 0.9740 | N/A |
| Visiting | OL v P3 | 0.3119 | N/A | 0.9528 | 0.9015 |
|  | OL v IG2 | 0.2388 | 0.3841 | 0.6678 | N/A |
|  | P3 v IG2 | 0.9985 | N/A | 0.9275 | N/A |
| Bouncing | OL v P3 | 0.1818 | N/A | 0.9674 | 0.8342 |
|  | OL v IG2 | 0.0961 | 0.3402 | 0.6559 | N/A |
|  | P3 v IG2 | 0.9897 | N/A | 0.8978 | N/A |
| Resting | OL v P3 | 0.2797 | N/A | 0.9999 | 0.2268 |
|  | OL v IG2 | 0.3265 | 0.5880 | 0.8416 | N/A |
|  | P3 v IG2 | 0.9997 | N/A | 0.8657 | N/A |

**Additional Table 6. Within treatment comparisons (p-value) of total activity split into four behavioural modes (swooping, visiting, bouncing and resting) on four ITNs (Untreated net = UT, Olyset Net = OL, PermaNet 3.0 = P3, Interceptor G2 = IG2) between four mosquito strains. Resistant mosquito strains are denoted in *italics*.**

| **Behaviour** | **Strain comparison** | **ITN** | | | |
| --- | --- | --- | --- | --- | --- |
|  |  | **UT** | **OL** | **P3** | **IG2** |
| Swooping | Kisumu v N’gousso | 0.0950 | 0.4483 | N/A | 0.9978 |
|  | Kisumu v *VK7* | 0.0010 | 0.4572 | 0.6651 | 0.8395 |
|  | Kisumu v *Banfora* | 0.0640 | 0.0879 | 0.9477 | N/A |
|  | N’gousso v *VK7* | 0.4166 | 0.9999 | N/A | 0.7475 |
|  | N’gousso v *Banfora* | 0.9984 | 0.8000 | N/A | N/A |
|  | *VK7* v *Banfora* | 0.5157 | 0.8484 | 0.9748 | N/A |
| Visiting | Kisumu v N’gousso | 0.0352 | 0.7484 | N/A | 0.9844 |
|  | Kisumu v *VK7* | 0.0248 | 0.6266 | 0.9997 | 0.9377 |
|  | Kisumu v *Banfora* | 0.5026 | 0.7043 | 0.9989 | N/A |
|  | N’gousso v *VK7* | 0.9994 | 0.9946 | N/A | 0.9997 |
|  | N’gousso v *Banfora* | 0.5523 | 0.9998 | N/A | N/A |
|  | *VK7* v *Banfora* | 0.4796 | 0.9980 | 0.9999 | N/A |
| Bouncing | Kisumu v N’gousso | 0.3291 | 0.9915 | N/A | 0.7701 |
|  | Kisumu v *VK7* | <0.0001 | 0.9997 | 0.5171 | 0.7801 |
|  | Kisumu v *Banfora* | 0.0014 | 0.9985 | 0.8952 | N/A |
|  | N’gousso v *VK7* | 0.0032 | 0.9822 | N/A | 1.000 |
|  | N’gousso v *Banfora* | <0.0001 | 0.9697 | N/A | N/A |
|  | *VK7* v *Banfora* | <0.0001 | 0.9999 | 0.9669 | N/A |
| Resting | Kisumu v N’gousso | 0.1368 | 0.5673 | N/A | 0.3099 |
|  | Kisumu v *VK7* | 0.0004 | 1.0000 | 0.3601 | 0.8771 |
|  | Kisumu v *Banfora* | 0.8988 | 0.9949 | 0.8801 | N/A |
|  | N’gousso v *VK7* | 0.0668 | 0.5917 | N/A | 0.7962 |
|  | N’gousso v *Banfora* | 0.1368 | 0.3588 | N/A | N/A |
|  | *VK7* v *Banfora* | 0.0001 | 0.9891 | 0.1704 | N/A |

**Additional Table 7. Mean total number of bed net contacts [95% CI], mean total contact duration [95% CI] and maximum number of mosquitoes seen in one frame of video recording. Resistant mosquito strains are denoted in *italics*.**

| **ITN** | **Strain** | **Replicates** | **Mean total number of contacts [95% CI]** | **Mean total contact duration (seconds) [95% CI]** | **Maximum number of mosquitoes** |
| --- | --- | --- | --- | --- | --- |
| UT | Kisumu | 5 | 74885  [53016.58, 96753.42]] | 9044.20  [7723.32, 10202.13] | 29 |
|  | N’gousso | 4 | 62162.25  [39731.46, 84593.04] | 8254.66  [5049.89, 11459.43] | 17 |
|  | *VK7* | 4 | 41811.25  [31737, 51885.5] | 5783.19  [3589.78, 7976.59] | 14 |
|  | *Banfora* | 4 | 80824  [43804.85, 117843.20] | 11005.31  [7866.99, 14143.62] | 14 |
| OL | Kisumu | 6 | 6169.17  [2521.05, 9817.29] | 622.03  [238.49, 1005.57] | 16 |
|  | N’gousso | 5 | 4531.17  [3175.86, 5886.47] | 342.37  [256.65, 428.09] | 10 |
|  | *VK7* | 6 | 7393.2  [3465.90, 11320.50] | 682.53  [532.04, 833.02] | 9 |
|  | *Banfora* | 6 | 7413.5  [3695.42, 11167.58] | 787.19  [268.22, 1306.16] | 14 |
| P3 | Kisumu | 6 | 10909.33  [3149.47, 18669.20] | 1929.23  [187.19, 3671.27] | 27 |
|  | *VK7* | 5 | 6219  [2576.16, 9861.84] | 1164.04  [[736.89, 1591.20] | 9 |
|  | *Banfora* | 3 | 14772  [1453.14, 28090.86] | 1668.92  586.24, 2751.60] | 8 |
| IG2 | Kisumu | 6 | 12759.5  [6312.24, 19206.76] | 2236.16  [1434.93, 3037.40] | 14 |
|  | N’gousso | 6 | 6686.5  [2384.28, 10988.75] | 1822.44  [971.85, 2673.03] | 16 |
|  | *VK7* | 5 | 10488  [4366.25, 16609.75] | 1587.40  [853.88, 2320.90] | 11 |

**Additional Table 8. Within strain statistical comparisons (p value) of total number of net contacts for susceptible (Kisumu and N’gousso) and resistant (VK7 and Banfora) mosquitoes between ~~three ITNs~~four nets (UT = untreated, OL = Olyset Net, P3 = PermaNet 3.0, IG2 = Interceptor G2).** **Resistant mosquito strains are denoted in *italics*.**

| **Net comparison** | **Strain** | | | |
| --- | --- | --- | --- | --- |
|  | **Kisumu** | **N’gousso** | ***VK7*** | ***Banfora*** |
| **UT v OL** | <0.0001 | <0.0001 | <0.0001 | <0.0001 |
| **UT v P3** | <0.0001 | N/A | <0.0001 | <0.0001 |
| **UT v IG2** | <0.0001 | <0.0001 | <0.0001 | N/A |
| **OL v P3** | 0.7873 | N/A | 0.9966 | 0.6402 |
| **OL v IG2** | 0.5684 | 0.9741 | 0.9445 | N/A |
| **P3 v IG2** | 0.9833 | N/A | 0.8689 | N/A |

***Additional Table 9. Within treatment statistical comparisons (p value) of total number of net contacts for four nets between four mosquito strains.*** ***Resistant mosquito strains are denoted in italics.***

| **Strain comparison** | **ITN** | | | |
| --- | --- | --- | --- | --- |
|  | **Untreated** | **Olyset Net** | **PermaNet 3.0** | **Interceptor G2** |
| **Kisumu v N’gousso** |  | 0.9883 | N/A | 0.6322 |
| **Kisumu v *VK7*** | <0.0001 | 0.9957 | 0.8151 | 0.9738 |
| **Kisumu v *Banfora*** |  | 0.9948 | 0.9250 | N/A |
| **N’gousso v *VK7*** | 0.0095 | 0.9496 | N/A | 0.8914 |
| **N’gousso v *Banfora*** | 0.0202 | 0.9414 | N/A | N/A |
| ***VK7* v *Banfora*** | <0.0001 | 1.0000 | 0.5473 | N/A |

**Additional Table 10. Within strain comparisons (p-value) of total duration of net contact for susceptible (Kisumu and N’gousso) and resistant (VK7 and Banfora) mosquitoes between three ITNs (OL = Olyset Net, P3 = PermaNet 3.0, IG2 = Interceptor G2).** **Resistant mosquito strains are denoted in *italics*.**

| **Net comparison** | **Strain** | | | |
| --- | --- | --- | --- | --- |
|  | **Kisumu** | **N’gousso** | ***VK7*** | ***Banfora*** |
| **UT v OL** | <0.0001 | <0.0001 | <0.0001 | <0.0001 |
| **UT v P3** | <0.0001 | N/A | <0.0001 | <0.0001 |
| **UT v IG2** | <0.0001 | <0.0001 | <0.0001 | <0.0001 |
| **OL v P3** | 0.1265 | N/A | 0.8889 | 0.6018 |
| **OL v IG2** | 0.0373 | 0.0617 | 0.5123 | N/A |
| **P3 v IG2** | 0.9514 | N/A | 0.9088 | N/A |

**Additional Table 11. Within treatment comparison (p-value) of total net contact duration for three ITNs between four mosquito strains.** **Resistant mosquito strains are denoted in *italics*.**

| **Strain comparison** | **ITN** | | | |
| --- | --- | --- | --- | --- |
|  | **Untreated** | **Olyset Net** | **PermaNet 3.0** | **Interceptor G2** |
| **Kisumu v N’gousso** |  | 0.9567 | N/A | 0.8908 |
| **Kisumu v *VK7*** | 0.0001 | 0.9994 | 0.5914 | 0.7097 |
| **Kisumu v *Banfora*** | 0.0252 | 0.9938 | 0.9829 | N/A |
| **N’gousso v *VK7*** | 0.0051 | 0.9310 | N/A | 0.9801 |
| **N’gousso v *Banfora*** | 0.0015 | 0.8683 | N/A | N/A |
| ***VK7* v *Banfora*** | <0.0001 | 0.9992 | 0.9006 | N/A |

**Additional Table 12. Percentage of contact duration in first the 10minutes of room scale tracking assay – within strain, between net differences.** **Resistant mosquito strains are denoted in *italics*.**

| **Net comparison** | **Strain** | | | |
| --- | --- | --- | --- | --- |
|  | **Kisumu** | **N’gousso** | ***VK7*** | ***Banfora*** |
| **UT v OL** | <0.0001 | <0.0001 | 0.9999 | 0.9965 |
| **UT v P3** | 0.0121 | N/A | 0.0547 | 0.8800 |
| **UT v IG2** | 0.0003 | 0.0108 | 0.9592 | N/A |
| **OL v P3** | 0.0626 | N/A | 0.0312 | 0.9302 |
| **OL v IG2** | 0.5533 | 0.0243 | 0.9327 | N/A |
| **P3 v IG2** | 0.6108 | N/A | 0.1253 | N/A |

**Additional Table 13. Percentage of contact duration in first 10mins of assay – within net, between strain differences Resistant mosquito strains are denoted in *italics*.**

| **Strain comparison** | **ITN** | | | |
| --- | --- | --- | --- | --- |
|  | **Untreated** | **Olyset Net** | **PermaNet 3.0** | **Interceptor G2** |
| **Kisumu v N’gousso** | 0.9829 | 0.7099 | N/A | 0.9489 |
| **Kisumu v *VK7*** | 0.9717 | <0.0001 | 0.8614 | 0.0004 |
| **Kisumu v *Banfora*** | 0.9996 | <0.0001 | 0.1913 | N/A |
| **N’gousso v *VK7*** | 0.8703 | <0.0001 | N/A | 0.0021 |
| **N’gousso v *Banfora*** | 0.9707 | <0.0001 | N/A | N/A |
| ***VK7* v *Banfora*** | 0.9884 | 0.9062 | 0.5609 | N/A |

**Additional Table 14. Average contact duration in first 10minutes – within strain, between net comparisons. Resistant mosquito strains are denoted in *italics*.**

| **Net comparison** | **Strain** | | | |
| --- | --- | --- | --- | --- |
|  | **Kisumu** | **N’gousso** | ***VK7*** | ***Banfora*** |
| **UT v OL** | 0.8368 | 0.0083 | 0.9488 | 0.0607 |
| **UT v P3** | 0.9476 | N/A | 0.7547 | 0.3962 |
| **UT v IG2** | 0.1146 | 0.9217 | 1.0000 | N/A |
| **OL v P3** | 0.9899 | N/A | 0.3730 | 0.9217 |
| **OL v IG2** | 0.0092 | 0.0199 | 0.9347 | N/A |
| **P3 v IG2** | 0.0223 | N/A | 0.7299 | N/A |

**Additional Table 15. Average contact duration in first 10minutess – within net, between strain comparisons.** **Resistant mosquito strains are denoted in *italics*.**

| **Strain comparison** | **ITN** | | | |
| --- | --- | --- | --- | --- |
|  | **Untreated** | **Olyset Net** | **PermaNet 3.0** | **Interceptor G2** |
| **Kisumu v N’gousso** | 0.3666 | 0.7884 | N/A | 0.6326 |
| **Kisumu v *VK7*** | 0.1950 | 0.2028 | 0.9356 | 0.0002 |
| **Kisumu v *Banfora*** | 0.9943 | 0.6882 | 0.7480 | N/A |
| **N’gousso v *VK7*** | 0.0054 | 0.6882 | N/A | 0.0075 |
| **N’gousso v *Banfora*** | 0.5587 | 0.8701 | N/A | N/A |
| ***VK7* v *Banfora*** | 0.1498 | 0.9818 | 0.9622 | N/A |

**Additional Table 16. Comparison (p-value) of average swooping speeds across 2hour assay within four different strains, between four different net treatments. Resistant mosquito strains are denoted in *italics*.**

| **Net comparison** | **Strain** | | | |
| --- | --- | --- | --- | --- |
|  | **Kisumu** | **N’gousso** | ***VK7*** | ***Banfora*** |
| **UT v OL** | 0.0226 | 0.4931 | 0.9972 | 0.2854 |
| **UT v P3** | 0.0937 | N/A | 0.9910 | 0.2929 |
| **UT v IG2** | 0.0092 | 0.8099 | 0.9995 | N/A |
| **OL v P3** | 0.9276 | N/A | 0.9996 | 0.9920 |
| **OL v IG2** | 0.9861 | 0.9345 | 0.9879 | N/A |
| **P3 v IG2** | 0.7756 | N/A | 0.9735 | N/A |

**Additional Table 17. Comparison of average swooping speeds across 2hour assay within four net treatments, between four strains. Resistant mosquito strains are denoted in *italics*.**

| **Strain comparison** | **ITN** | | | |
| --- | --- | --- | --- | --- |
|  | **Untreated** | **Olyset Net** | **PermaNet 3.0** | **Interceptor G2** |
| **Kisumu v N’gousso** | 0.0013 | 0.0173 | N/A | 0.1576 |
| **Kisumu v *VK7*** | 0.0240 | 0.9555 | 0.6271 | 0.9987 |
| **Kisumu v *Banfora*** | 0.0164 | 0.0736 | 0.0332 | N/A |
| **N’gousso v *VK7*** | 0.7782 | 0.0882 | N/A | 0.1414 |
| **N’gousso v *Banfora*** | 0.8472 | 0.9390 | N/A | N/A |
| ***VK7* v *Banfora*** | 0.9991 | 0.2601 | 0.3216 | N/A |

**Additional Table 18. Comparison of activity decay over time (p-value), within strain, between net treatment. Resistant mosquito strains are denoted in *italics*.**

| **Net comparison** | **Strain** | | | |
| --- | --- | --- | --- | --- |
|  | **Kisumu** | **N’gousso** | ***VK7*** | ***Banfora*** |
| **UT v OL** | 0.0023 | 0.8774 | 0.0128 | 0.1454 |
| **UT v P3** | 0.0020 | N/A | 0.0010 | 0.2103 |
| **UT v IG2** | <0.0001 | 0.1902 | 0.0387 | N/A |
| **OL v P3** | 1.000 | N/A | 0.8049 | 0.9987 |
| **OL v IG2** | 0.3361 | 0.4861 | 0.9708 | N/A |
| **P3 v IG2** | 0.3894 | N/A | 0.5401 | N/A |

**Additional Table 19. Comparison of activity decay over time (p-value), within net treatment, between strains. Resistant mosquito strains are denoted in *italics*.**

| **Strain comparison** | **ITN** | | | |
| --- | --- | --- | --- | --- |
|  | **Untreated** | **Olyset Net** | **PermaNet 3.0** | **Interceptor G2** |
| **Kisumu v N’gousso** | 0.0734 | 0.9965 | N/A | 0.9745 |
| **Kisumu v *VK7*** | 0.4510 | 0.2427 | 0.7543 | 0.0013 |
| **Kisumu v *Banfora*** | 0.9962 | 0.2987 | 0.5513 | N/A |
| **N’gousso v *VK7*** | 0.0021 | 0.3397 | N/A | 0.0047 |
| **N’gousso v *Banfora*** | 0.0609 | 0.4128 | N/A | N/A |
| ***VK7* v *Banfora*** | 0.6268 | 0.9969 | 0.9675 | N/A |
